# Multiple Correspondence and Hierarchical Cluster Analyses for the Profiling of Fresh Apple Customers Using Data from Two Marketplaces

**DOI:** 10.3390/foods9070873

**Published:** 2020-07-03

**Authors:** Masoumeh Bejaei, Margaret A. Cliff, Amritpal Singh

**Affiliations:** 1 Summerland Research and Development Centre, Science and Technology Branch, Agriculture and Agri-Food Canada, Summerland, BC V0H 1Z0, Canada; Amritpal.Singh@Canada.ca; 2 Food Nutrition and Health, Faculty of Land and Food Systems, The University of British Columbia, Vancouver, BC V6T 1Z4, Canada; Margaret.Cliff@ubc.ca

**Keywords:** multiple correspondence analysis, hierarchical cluster analysis, market segment, customer profile, apple cultivar, consumer survey

## Abstract

Purchase behavior and preferences for consumers of fresh apples were investigated using a consumer survey conducted at a special-event apple market. Survey respondents were asked to list apple cultivars they had purchased at the retail market and the special-event market. The special-event market offered many uncommon cultivars packed in clear plastic bags with a fixed weight and price. Respondents were also asked to identify their reasons for selection of each apple cultivar and answer demographic questions. A total of 169 customers completed the survey. Profiles of customers were identified using multiple correspondence analysis (MCA) and hierarchical cluster analysis (HCA), and the impact of the change in available apple cultivars on consumers’ purchase behavior was explored. Consumers primarily indicated four main reasons in the selection of their apples: visual appearance, previous experience, taste/aroma, and texture. The first two reasons, evaluated before eating an apple, were loaded on the first MCA dimension, while the last two reasons (i.e., eating quality) were loaded on the second dimension in data from both marketplaces. HCA identified five classes of customers in both markets, and results indicated that similar market segments existed within the two marketplaces, regardless of the availability of apple cultivars.

## 1. Introduction

The five apple producing provinces in Canada are British Columbia, Nova Scotia, New Brunswick, Ontario, and Quebec. Apples are one of the staple fruits in most Canadian grocery stores, and several domestic and imported apple cultivars are available year-round (e.g., Ambrosia, Honeycrisp, Gala, McIntosh, Red Delicious, Granny Smith, and Golden Delicious). Availability of fresh apple for consumption on a per person basis has slightly decreased in the last few decades [1]. Knowledge about the customer profiles will enable the industry and the breeding programs to select new apple cultivars considering given specific target markets, in order to improve the long-term economic viability of the Canadian apple industry.

Customers consider several sensory and non-sensory factors in the purchase of fresh fruits [2,3]. Some may value the nutritional content of fruits and vegetables and their health benefits [4] while others may purchase fruits on the consideration of the products’ intrinsic or extrinsic characteristics [5,6,7].

Previous studies [6,7,8] investigated the importance of fruit quality attributes (e.g., taste, size, color, and texture) on customers’ liking of fresh apples. In a market segment study focused on food safety (pesticide usage), price, fruit quality (level of damage on the apple), and compliance with food safety regulations for Red Delicious apples, Baker [2] identified four consumer segments: 42% of customers were “safety seekers”, 36% were “balanced buyers” (balanced concerns for all factors), 9% were “price pickers”, and 13% were “perfect produce buyers” (focused on fruit quality). This research demonstrated the diversity among customers and the importance for breeding programs, marketers, and retailers to understand the needs of their consumers to develop and supply products that will be successful in the marketplace.

The integrated effect of intrinsic food characteristics (e.g., color, taste, aroma, and texture) and extrinsic factors (e.g., packaging, price, and location of purchase or production) on the sensory perceptions of consumers has been discussed by Wang et al. [9]. They emphasized the importance of combining the intrinsic and extrinsic influences in evaluating specific sensory attributes of food products.

A number of consumer research studies have used the combination of multiple correspondence analysis (MCA) and hierarchical cluster analysis (HCA) to identify market segments of products by investigating interrelationships among categorical variables associated with consumers [10]. By studying consumer purchase behavior and identifying consumer profiles and market segments, the industry would be able to more successfully meet the needs of their consumers, independent of specific cultivar availability in each market. This is particularly important for the apple industry, where different fruit cultivars might be available at different markets during the year. Furthermore, knowledge of market segments would allow the industry to identify consumers that would be more willing to try new cultivars [11], on the basis of their interest/motivation to purchase apples with superior sensory attributes.

The current study was undertaken to acquire information about customers’ purchase behavior to assist with the appropriate selection of new cultivars by the breeding programs and identification of the mix of cultivars in specific markets by marketers, in order to optimally meet the needs of consumers. Thus, the main objectives of this study were twofold: (1) to identify fresh apple market segments and customer profiles, and (2) to investigate potential changes in the purchase behavior of the same customers when shopping for apples in two different marketplaces (i.e., retail market and special-event market) in which different apple cultivars were available for purchase.

## 2. Materials and Methods

### 2.1. Study Location

The University of British Columbia (UBC) Apple Festival is an annual event held by the non-profit organization called Friends of the Garden (FOGs) at UBC Botanical Garden (https://botanicalgarden.ubc.ca/about/friends-of-the-garden/). This festival brings together a diverse group of consumers, not only from the university campus but from the greater metropolitan region, for a two-day event related to apples. The festival incorporates a range of activities, all related to apples, including an exhibition, an apple cultivar tasting, and an apple market. At the apple market, FOGs have offered many uncommon (new, heritage) as well as a few readily available cultivars for sale for the last 28 years. In 2019, they listed 49 different cultivars for sale, some in limited supply. Eleven apple cultivars (Table 1) were selected to be included in this study because of the higher frequency of their purchase. The design of the apple sale section at the special-event market was similar to a field experiment set up [12] in which customers enter the market, browse the diverse products, select product(s) for purchase, and pay for their purchases using their own money. Many extrinsic factors were controlled as all apples sold at the festival were grown in British Columbia (BC), packaged in 1.4 kg clear plastic bags, and priced at $7/bag. In addition, information about the intrinsic characteristics of apples were either observed by the customers (e.g., skin color, size, and shape) or presented to customers using descriptive signs for each cultivar (Table 1). Each sign included information regarding the cultivar and was mounted directly above the bags of fruit at the special-event market.

The past purchase behavior of the customers in the retail market was investigated in addition to their purchases at the special-event market to be able to identify customer profiles in two marketplaces and evaluate the impact of the changes in the market settings and available cultivars on the purchase behavior of customers.

In addition to sensory characteristics for the cultivars (Table 1), FOGs also included information about the “usage”, “annual harvest timing”, “potential storage duration”, “best eating quality timing”, “origin”, and “ancestry” for each cultivar (data not shown).

### 2.2. Questionnaire Design

A one-page paper questionnaire (Figure S1) was designed to study customers’ behavior, attitudes, and preferences for fresh apple purchases. Ethics approval for the research was obtained from the Agriculture and Agri-Food Canada (AAFC) Human Research Ethics Committee, and communicated to the UBC Behavioural Research Ethics Board.

Customers were intercepted as they were exiting the special-event market, and were invited to participate in the study. Respondents were asked to identify the apple cultivars that they purchased in the retail market in the last 30 days prior to the survey, and the apple cultivars that they bought at the special-event market. They were also asked to give the reason(s) for their selection of each cultivar using seven choice options: visual appearance (e.g., skin color, shape, size), texture (e.g., crispness, hardness, juiciness), taste/aroma (e.g., fruitiness, flavor), health benefits (e.g., nutritional benefits), environmental concerns (e.g., organic, local, sustainable), price, previous experience, and other (requested to specify).

Respondents were also asked three categorical demographic questions (gender, age, and ethnicity), and one apple-consumption frequency question, evaluated on a 5-point scale where 1 = daily and 5 = once/month or less.

### 2.3. Data Collection

One experimenter collected data from attendees at the UBC Apple Festival over the two-day event (17–18 October 2019). FOGs volunteers assisted in the data collection process by inviting the festival attendees exiting the special-event market to participate in the survey.

### 2.4. Inclusion of External Data into Datasets

External data related to the fruit visual and sensory attributes (i.e., skin color and flavor) were either obtained by observing and tasting the retail market apples at the AAFC Sensory Evaluation and Consumer Research laboratory in Summerland over years or by the information presented by FOGs at the point of sale at the special-event market (Table 1). Flavor of apples purchased were classified in three categories: sweet, sweet-tart (or sub acid), and tart, on the basis of categories typically used in the apple industry.

### 2.5. Data Analysis

The responses from the paper ballots were assigned with an individual ID and the data were entered in Qualtrics Software (Qualtrics, Provo, UT, USA) before converting into a JMP dataset (JMP, Version 15.0.0; SAS Institute Inc., Cary, NC, USA) for statistical analysis.

The analysis was limited to the apple cultivars with at least 10 respondents (regardless of their ethnicities) who reported purchasing them either in the retail market or at the special-event market. Respondents were able to report multiple apple cultivars in each market.

The data were organized in two formats [13]: wide or multiple response format in which aligned responses of multiple apple cultivars listed by a respondent were entered across several columns but treated as one grouped response (i.e., one row per respondent), and long or multiple response by ID format in which multiple responses across rows that have the same ID values (i.e., one row per cultivar per respondent). The wide dataset was utilized to study the relationship between the variables that had one response per participant (e.g., age and gender of the respondents) using chi-square (χ^2^) tests, whereas the long dataset was used in analyzing the customer purchase behavior and preferences using MCA and HCA.

Instead of calculating relationships pair-wise, the inter-relationships among all the variables were evaluated and visualized using MCA. MCA is a non-parametric statistical test developed for categorical data [14]. It reduces the dimensionality of data, similar to factor analysis and principle component analysis in parametric tests. Detailed descriptions of MCA and HCA are explained by Ganiere et al. [10].

#### 2.5.1. Multiple Correspondence Analysis (MCA)

Considering descriptive results and the frequencies of the occurrence of reasons for the purchase of apple cultivars, we selected four variables (visual appearance, texture, taste/aroma, and previous experience) to be analyzed using MCA. These factors were all binomial variables, where consumers’ responses were either yes/no. The analyses were conducted separately on the retail market and special-event market data.

MCA results in a minimum number of latent variables (or components) with linear combinations of the original variables that are independent from each other. MCA makes it possible to observe the expression of some concepts that are not directly observable in the original variables. It converts deviations from the independence model in the contingency table (using the chi-square metric) into distances.

The *p* eigenvalues in MCA are extracted from a matrix developed from the Burt’s matrix and the latent variables (components) reproduce, in descending order, the highest variation (inertia) among the observations in the Burt’s matrix (as also discussed by Salis et al. [15]). The total number of eigenvalues, *p,* is determined by considering the dimension of the dataset, as indicated in Equation (1).

*p* = *n* − *k* (1)

where *k* is the number of variables included in the MCA and *n* the total number of possible values that the variables may assume. The categories with larger sample sizes are closer to the center of the map in MCA and lower percentage of data variability is explained by them.

#### 2.5.2. Hierarchical Cluster Analysis (HCA)

The resultant dimensions (or components) obtained from MCA (for each consumer) were then utilized in the HCA, using Ward’s minimum variance method [16]. On the basis of this method, homogenous clusters of customers were identified with their common characteristics in order to create the profile of the cluster.

Using an appropriate measure of distance between pairs of observations, similar observations were grouped in one cluster while different clusters included distinct groups. A “bottom up” method is used in a hierarchical clustering of agglomerative type. Numerical variables (i.e., factorial or compositional scores generated by MCA) are considered, the Euclidean distance is used, while the Ward’s linkage criterion is used in structuring the hierarchy [16]. The objective of Ward’s linkage method is to group observations into a cluster to reduce variation within that cluster. Thus, an observation is included in a cluster if its inclusion in that cluster results in the least increase in the error sum of squares. The distance of the Ward’s method (*D_AB_*) is calculated using Equation (2).

(2) $$D_{AB}=\frac{{\left\|\bar{X_{A}}-\bar{X_{B}}\right\|}^{2}}{\frac{1}{N_{A}}+\frac{1}{N_{B}}}$$

where *A* and *B* represent the set of clusters; *N_A_* and *N_B_* are the number of observations in clusters *A* and *B*, respectively; $\bar{X_{A}}$ and $\bar{X_{B}}$ demonstrate the mean vectors representing clusters *A* and *B*; and ${\left\|\bar{X_{A}}-\bar{X_{B}}\right\|}^{2}$ shows the squared Euclidean distance between vectors $\bar{X_{A}}$ and $\bar{X_{B}}$.

## 3. Results and Discussion

### 3.1. Survey Participants

A total of 169 customers completed the survey. The gender, age, and ethnicity of the survey respondents were compared with BC population census information in Table 2 to evaluate if a representative sample of randomly selected customers participated in the survey.

There were male and female respondents from all age groups in this study; however, there were twice as many female respondents compared to male respondents. It is common in most food purchase related surveys to have more female respondents, since women are most often responsible for grocery shopping and willing to participate in grocery shopping-related surveys [18].

A chi-square test of independence revealed that there was no significant association between the gender (two levels: male and female) and age of the respondents (six age groups) in this study, χ^2^_(5, *N* = 149)_ = 3.68, *p* = 0.60. Data from the “other” or “unknown” category could not be included in the analysis, since the cell count did not meet the minimum requirement of five. The gender of the respondents was also independent from their frequency of consumption (χ^2^_(8, *N* = 168)_ = 3.99, *p* = 0.86) or their ethnicities (four groups) (χ^2^_(3, *N* = 150)_ = 1.55, *p* = 0.67).

The majority of the respondents ate apples 3–5 times/week (30.4%) or daily (35.7%), and 19.1% of them consumed apples once or twice/week, while 14.9% of the respondents ate apples only a few times/month or less. To investigate whether the frequency of consumption was independent from the demographic characteristics of the respondents, the last two frequency groups were merged to provide a large enough sample size in each group for the chi-square tests. Apple consumption frequencies were independent from the gender of the respondents (χ^2^_(3, *N* = 150)_ = 0.65, *p* = 0.86) or their ethnicity (χ^2^_(6, *N* = 156)_ = 4.11, *p* = 0.66).

The customers’ reasons for the selection of different apple cultivars and their information is investigated in the next sections.

### 3.2. Multiple Correspondence Analysis (MCA)

**Figure 1:**
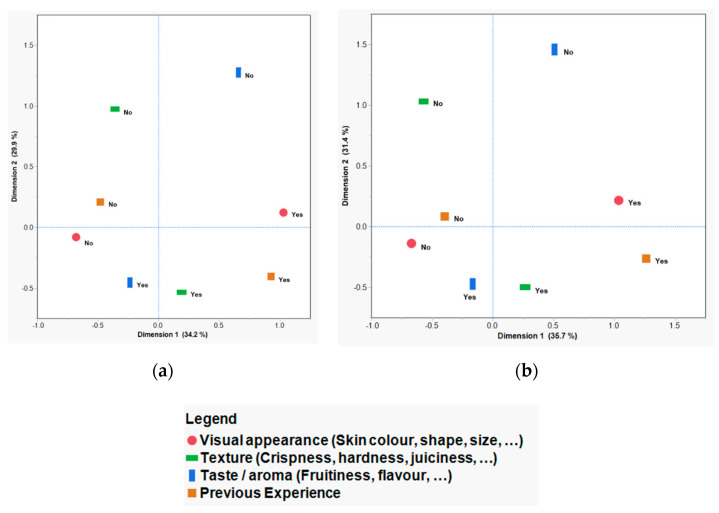
Multiple correspondence analysis (MCA) of retail market (**a**) and special-event market (**b**) data showing the two-dimensional plot (dimension 1 versus dimension 2), created using the four most important reasons for purchasing apples (visual appearance, texture, taste/aroma, and previous experience), as identified by consumers. Each reason was a binary variable with a yes/no response.

MCA was used to identify the profiles of the customers on the basis of the factors that were important to them for the purchase of fresh apples in different market settings. Figure 1 shows the two-dimensional MCA at the retail market (a) and special-event market (b), showing the four reasons (visual appearance, texture, taste/aroma, and previous experience) for selecting different apple cultivars.

The MCA plots (Figure 1a,b) were similar, indicating that availability of different and diverse cultivars at two marketplaces (i.e., retail market and special-event market) did not impact (change) the factors associated with the purchase decision making process. The two-dimensional MCA plots for the retail market (Figure 1a) and the special-event market (Figure 1b) data explained 64.2% and 67.1%, respectively, of the total inertia. The first three dimensions explained above 85% of the data, while all the variation (100%) was explained by the four dimensions in both market settings. Data from all four dimensions were utilized in the HCA to determine the customer profiles and clusters.

The majority of the respondents selected taste/aroma (three-quarters) and texture (two-thirds) as the most important factors in the purchase of apples in both marketplaces. Stow [19] also reported these two attributes as the main factors considered by apple customers. Fewer customers considered visual attributes (39%) and previous experience (34% at the retail market and 24% at the special-event market) in the selection of their apples.

#### 3.2.1. First Dimension in MCA

The first dimension in MCA accounted for about one-third of the inertia in both market settings, and was heavily loaded with two variables, visual appearance (i.e., “look”) and previous experience (i.e., “familiarity”). These purchase reasons were those that can be evaluated at first sight without eating the fruit, regardless of the eating quality of the fruit or the cultivars available.

The individuals who considered both of these purchase reasons (i.e., yes response) were plotted in the right side of the horizontal factor axis and the ones who did not consider these purchase reasons (i.e., no response) in the selection of their apples were at the left side of the axis (Figure 1) for both marketplaces. More than two-thirds of customers’ responses to the importance of “look” and “familiarity” reasons were the same (especially customers with a European background). Customers with different ethnicities responded differently to these questions. Visual appearance of apples was considered more important by customers with West Asian and South Asian backgrounds, while previous experience was selected more often by the respondents with a European background as the reason for the purchase of apples.

#### 3.2.2. Second Dimension in MCA

The second dimension in MCA also represented about one-third of the inertia in both marketplaces. Taste/aroma and texture variables were loaded heavily in this axis, and the majority of customers considered both of these reasons in their purchase decisions. They are both consumption-related sensory attributes that customers do not perceive directly at the time of purchase, but have expectations on the basis of their previous experience with the same or similar products or presented information at the time of purchase [20].

Textural sensory attributes were selected more often by the customers of European, West Asian, and South Asian backgrounds as the reason for the purchase of apples. In addition, more respondents with non-European backgrounds selected the taste/aroma (or flavor) attribute as the reason for selection of their apples, compared to the customers from European background.

About 1/4 of respondents with a European background purchased sweet-tart apples during the retail market purchase, while only 1/10th of customers with non-European backgrounds bought apples from this taste category at the same period of time. In contrast, 80% of the respondents with Asian and Southeast Asian backgrounds preferred sweet apples at the retail market compared to the 57.9% of respondents with European backgrounds. Even though the purchase of the sweet-tart apples increased at the special-event market due to the availability of uncommon (new) cultivars, such as Salish, the sweet-tart cultivars were still preferred more by customers of European rather than non-European backgrounds.

About one-third of the sweet-tart apples that customers with Asian and Southeast Asian backgrounds purchased during the special-event market were Honeycrisp; however, the contrast between its name and actual taste could have impacted the purchase of this apple by customers with Asian and Southeast Asian backgrounds, whom on the basis of the result of the current study prefer the sweet taste in apples. The name of the cultivar may also impact the sensory perceptions of the customers, as discussed by Rickard et al. [7] and Deliza and MacFie [21]. This topic requires further investigation.

### 3.3. Hierarchical Cluster Analysis (HCA) to Identify Market Segments

Data from all four dimensions of MCA (data not shown) were utilized in HCA and evaluated separately on data from two marketplaces. Customers were then grouped into five classes on the basis of the results of the HCA. Each class was assigned with a name (“name”), and results from the two marketplaces were compared with each other. The characteristics of each cluster were described using the consumers’ choice of cultivars, reasons for the selection of the cultivars, age, gender, and ethnicity, as well as their frequency of apple consumption.

The five classes of customers that were identified in the retail market were very similar to those identified in the special-event market. The only difference was in the order of one of the classes in which the third group of customers in the retail market were the first group at the special-event market. This resulted in a change in the order of the first three classes of customers in two datasets. Classes 1, 2, 3, 4, and 5 in the retail market, and 2, 3, 1, 4, and 5 in the special-event market, were named as “better eating quality seekers”, “better eating quality seekers of familiar or good-looking apples”, “taste lover buyers”, “perfect product seekers”, and “cultivar-loyal buyers”, respectively. The names of customer classes were developed through considering the frequencies of selection of the top four purchase reasons (i.e., visual appearance, texture, taste/aroma, and previous experience) by five different classes of customers at the retail and special-event markets (as reported in Table 3).

**Figure 2:**
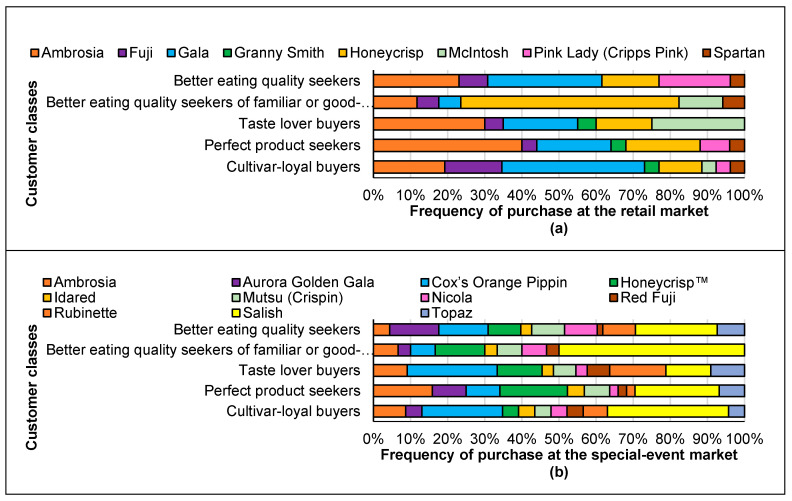
Apple cultivars purchased by five different classes of customers at the retail market (**a**) and special-event market (**b**).

Figure 2 shows the apple cultivars selected by each class of customers at the retail market (a) and the special-event market (b). The characteristics of the five classes of customers are described below.

**Figure 3:**
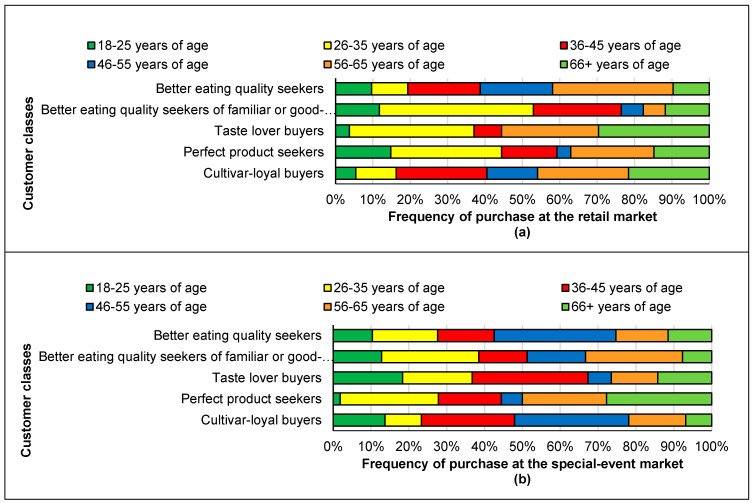
Age of the respondents in the different classes of consumers purchasing apples at the retail market (**a**) and special-event market (**b**).

The age distribution of the respondents was different from the different classes of customers (Figure 3).

**Figure 4:**
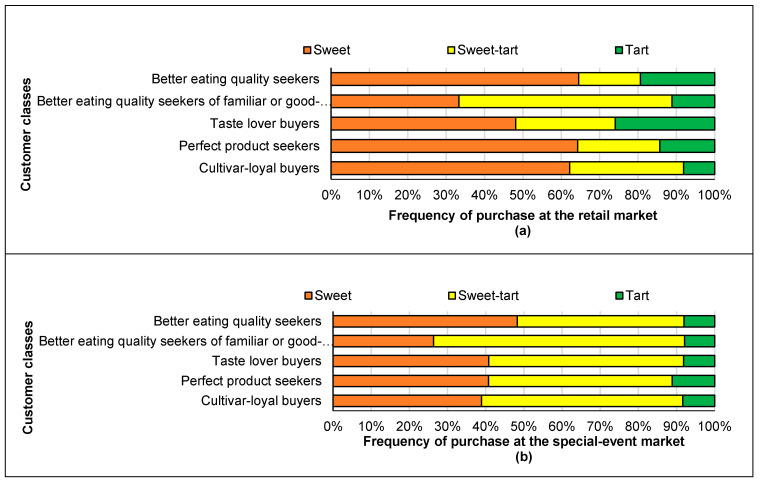
Taste category (sweet, sweet-tart, tart) of apples preferred by different classes of consumers at the retail market (**a**) and special-event market (**b**).

Figure 4 shows the taste category (sweet, sweet-tart, tart) of the apples preferred by different classes of consumers. More diversity existed in the cultivars offered at the event with more sweet-tart apple choices compared to the retail market.

#### 3.3.1. Better Eating Quality Seekers

“Better eating quality seekers” represented 22.0% of the respondents in the retail market data and 28.8% of respondents in the special-event market data. These consumers selected their apples 100% on the basis of their eating qualities, including taste/aroma and texture, without any influence of the visual characteristics or previous experience.

Customers in this category purchased more Pink Lady (Cripps Pink) apples compared to the other customers at the retail market, and also enjoyed Ambrosia and Gala apples. Respondents in this class purchased more Aurora Golden Gala, Mutsu, and Nicola apples compared to the other groups of customers at the special-event market, while enjoying many of the other cultivars as well.

About one-third of individuals in this class considered environmental concerns in selecting their apples in both marketplaces and it has been reported that these concerns may result in purchasing more organic products [22]. Nevertheless, health benefits and price were not in the minds of these individuals when selecting their apples.

Three-quarter of these customers consumed at least three apples per week. The majority of the respondents in this class were 46 years of age or older, and three-quarters had a European ethnicity background in both the retail and special-event markets datasets. One-third of customers with an Asian background were in this class in the retail market data, and 42.9% of the customers with an Asian background were in this class in the special-event market data. Half of the customers with a West Asian background were also in this class at the special-event market.

These respondents bought more yellow skin apples compared to the other classes of customers, especially at the special-event market (21.6% of them), although the proportion of yellow skin apples purchased was much lower in general compared to the bi-color apple purchased.

About two-thirds of the apples purchased in this class at the retail market and about half of the apples bought at the special-event market in this class were considered sweet. As a result, this group mostly prefers sweet apples. About one-fifth of individuals in this class also purchased tart apples in the retail market.

In summary, this multicultural class of middle age and older customers includes about a quarter of the market and is a target market for future new premium apple selections. Regardless of the skin color and visual appearance characteristics, these new selections would need to have superior eating quality, but could be either sweet or tart.

#### 3.3.2. Better Eating Quality Seekers of Familiar or Good-Looking Apples

“Better eating quality seekers of familiar or good-looking apples” represented 12.8% of the respondents in the retail market data and 12.8% of respondents in the special-event market data. At the retail market, they selected their apples on the basis of the taste/aroma (100%), texture (72.2%), and previous experience (100%) attributes. At the special-event market, many cultivars were uncommon so instead of the familiarity factor, they considered the visual appearance (100%), taste/aroma (100%), and texture (89.7%) in their purchase decision. For these respondents, not only a better eating quality was important but they also needed to know (have experience with) the cultivar (at the retail market) or like its visual appearance (at the special-event market).

More than half of these customers (58.8%) bought Honeycrisp and 12% of them purchased McIntosh apples at the special-event market. These customers did not buy Ambrosia or Gala apples at the retail market particularly frequently. Half of these consumers purchased Salish apple and 13% of them still purchased Honeycrisp apple at the special-event market. In this class, the majority of customers preferred sweet-tart apples in both retail and special-event markets.

More than a quarter of these respondents considered health benefits of apples and environmental concerns in selecting their apples in both market settings. For about one-third of them, price at the retail market was also an important factor.

In general, individuals in this class were younger than other classes within the same dataset, especially among retail market customers. About half of the retail market class 2 individuals were under 35 years of age and 38.4% of the special-event market class 3 customers were in the same age range.

Similar to all classes of customers, the majority of the respondents were from a European background; however, above 20% of retail market customers and about 40% of the special-event market customers in this class were from non-European ethnicities (including 20% Asian or Southeast Asian background customers at the special-event market).

In summary, this class of younger customers included about 1/10th of the market and is a target market for premium bicolored apples with superior eating quality from sweet-tart taste category. These consumers tend to stay with the cultivars they already know unless the new cultivars are also superior in visual appearance in addition to having better eating quality.

#### 3.3.3. Taste Lover Buyers

“Taste lover buyers” represented 19.2% of the respondents in the retail market data and 16.0% of respondents in the special-event market data. In both markets, individuals assigned to this class selected their apples on the basis of taste/aroma only (and only 23% of them considered the visual appearance in the selection of apples at the retail market).

At the retail market, one-third of these individuals purchased Ambrosia, one-fifth bought Gala, and one-quarter purchased McIntosh apples. They did not purchase many Honeycrisp apples in the retail market. Considering the diversity of the cultivars at the special-event market, these customers preferred Cox’s Orange Pippin (24.2%), Rubinette (12.1%), Honeycrisp (12.1%), Salish (12.1%), Ambrosia (9.1%), and Topaz (9.1%) apple cultivars.

Above 80% of consumers purchased bicolored apples and a small percentage of the respondents in this class purchased green apples (3.8%) in the retail market.

Health benefits were not important for these customers, especially while shopping at the special-event market, and these individuals were not concerned about the environmental issues or price of the products.

The majority of the individuals in this class consumed at least three apples per week. Although the majority of customers in both markets were from European backgrounds, this class also included half of the customers from Southeast Asian backgrounds in both markets. More individuals of 36–45 years of age were in this class in the special-event market compared to their population in the other classes. In general, the individuals assigned to this class in the special-event market were younger consumers compared to the other classes.

About half of the individuals in this class purchased sweet apples and about a quarter of individuals selected tart apples at the retail market. For these customers, taste was the only important factor, and thus they selected either sweet or tart apples much more than sweet-tart apples at the retail market. However, at the special-event market, about half of them selected sweet-tart apples, 40.8% bought sweet apples, and the rest of consumers preferred tart apples. This could be related to the fact that previous experience was not an important reason for this group in the purchase of apples, and as a result they selected diverse cultivars of apples. Looking at the cultivars purchased, it was evident that these consumers purchased more Cox’s Orange Pippin (a sweet-tart apple) than other customers. At the point of sale, Cox’s Orange Pippin apples were identified as good for fresh eating as well as cider production. Since cultivars for cider production are known to be astringent, this characteristic might have been the appeal for the “taste lover buyers”. In addition, Cox’s Orange Pippin apple is widely known to have a unique flavor.

In summary, this class of younger customers consisted of one-fifth of the market and is a target market for apples with superior taste. They may be willing to try new cultivars if the taste is better, and they do not have specific preferences in regard to the visual attributes (e.g., skin color) as long as the taste is great.

#### 3.3.4. Perfect Product Seekers

“Perfect product seekers” represented 19.9% of the respondents in the retail market data and 18.0% of respondents in the special-event market data. At the retail market, they purchased their apples through considering all four reasons: visual appearance (100%), texture (100%), taste/aroma (100%), and previous experience (53.6%). At the special-event market, the individuals assigned to this class of customers also selected their apples by considering all four factors: visual appearance (63.6%), texture (85.5%), taste/aroma (100%), and previous experience (100%).

In the retail market, 40% of the respondents in this class purchased Ambrosia apples (the most Ambrosia apples purchased compared to the other classes), 20% Gala, and 20% Honeycrisp apples. Similar results were observed in the special-event market, and customers in this class also purchased the most Ambrosia (15.9% of them in the class) and Honeycrisp (18.2%) apples, and 9.1% of them bought Aurora Golden Gala and 22.7% Salish apples. These customers have stayed with well-known cultivars (regionally) in most cases.

Health concerns, environmental concerns, and price were more important for the customers from this class compared to the customers from other classes. About 70% of them eat apples at least three times per week.

In the retail market, one-third of the customers with Asian backgrounds were in this class and fewer (18%) of them were in this class in the special-event market. More customers with non-European backgrounds were in this class in the retail market compared to the event (39% versus 15%, respectively).

About 60% and 45% of the customers in this class at the retail and special-event markets, respectively, were younger than 45 years of age.

At the special-event market, 14.8% of the customers in this class purchased yellow skin apples. In the retail market, sweet apples were preferred by 64.3% of the customers in this class, while 14.3% of them purchased tart apples. At the special-event market, 40.7% of them still bought sweet apples, 11.1% of them purchased tart apples, and half of them preferred sweet-tart apples.

In summary, this multicultural class of younger customers consists of about one-fifth of the market, preferring well-known superior apple cultivars as they look for a perfect product. They are not an ideal group for the introduction of new apple selections at the earlier stages of commercialization, since these consumers tend to buy already well-established cultivars (i.e., they are laggards) and they will adopt the successful cultivars later [11].

#### 3.3.5. Cultivar-Loyal Buyers

“Cultivar-loyal buyers” represented 26.2% of the respondents in the retail market data and 24.5% of respondents in the special-event market data. This was the only class of customers who do not consider taste in selecting their apples. About 60% of them in both markets took into account the visual appearance of the apples and half of them considered texture of the apples, while about a quarter of them selected previous experience as an important factor.

About half of these customers bought Gala (38.5%) or Fuji (15.4%) apples in the retail market, and 19.2% of them purchased Ambrosia apples. At the event, one-third of them purchased Salish and 21.7% of them bought Cox’s Orange Pippin. The selected cultivars were mostly traditional, heritage, or BC-bred apple cultivars.

Health concerns, environmental concerns, and price were not the most important factors for them compared to the importance of these factors for customers from the other classes. More than three-quarters of these individuals consumed at least three apples per week.

These class 5 groups had the least customers with non-European background (less than one-fifth) compared to the other classes of customers of both markets, and they were mainly customers with European backgrounds. At the retail market, 46% of these customers were older than 55 years of age, while 22% of them were in that age range at the special-event market. At the special-event market, about one-third of the customers were from the 46–55 year age group.

At the retail market, 62.2% of them bought sweet apples and 29.7% of them purchased tart apples. At the special-event market, 38.9% of them bought sweet apples and 52.8% sweet-tart apples.

In summary, these middle-aged European background customers consisted of about one-quarter of the market. They often considered the visual appearance of the fruit without paying that much attention to the taste in the selection of their apples. They preferred to purchase established traditional, heritage, or BC-bred apple cultivars. This could indicate that they may not try a new selection if there is a well-known superior looking apple cultivar at the market.

## 4. Conclusions

A survey was conducted to gather information regarding consumers’ purchase behavior of apples at a special-event and retail markets. The special-event market was similar to an experimental market, where uncommon (new, heritage) and readily-available apple cultivars were sold. The age and ethnicity of the participants matched those of British Columbia residents, and respondents answered questions about their purchase behavior in two marketplaces. The application of MCA and HCA on retail market and special-event market data (separately) in this study resulted in the identification of five classes of apple customers with similar customer profiles in both datasets. This indicated that these classes were independent of types of apple cultivars sold in the market, since different apple cultivars were offered at these two markets. HCA identified five classes of customers in both markets (1) “better eating quality seekers”, (2) “better eating quality seekers of familiar or good-looking apples”, (3) “taste lover buyers”, (4) “perfect product seekers”, and (5) “cultivar-loyal buyers”. The first three classes of customers might be more willing to try specific new apple selections given that these consumers seek apples with superior sensory characteristics (visual appearance, taste/aroma, texture).

It should be noted that respondents in this research reported only the reasons that they were aware of (i.e., conscious reasons) for purchasing apples. There might, however, be other reasons that they were unaware of (i.e., unconscious reasons) that could influence their expectations, perceptions, and purchase of apples—such as apple name. Evaluation of such influences would require further research.

The apple breeding programs and apple industry could benefit from the information in this study in order to assist with estimating potential market opportunities for their new selections. Fruit retailers and marketers can also use the findings to identify the desirable apple cultivars for their customers.

## Figures and Tables

**Table 1:** Information presented by the Friends of Garden (FOGs) at the University of British Columbia (UBC) Apple Festival for the cultivars purchased frequently at the special-event market.

Name of the Cultivar	Sensory Characteristics
Ambrosia	Crisp, juicy, sweet, honeyed
Aurora Golden Gala	Extremely crisp, juicy, sweet, light, sometimes honey
Cox’s Orange Pippin	Crisp, aromatic, sweet, slightly acidic, classic English dessert
Honeycrisp	Very crisp and juicy, sub acid, ranges from mild to aromatic
Idared	Juicy, fine-grained, a bit tart
Mutsu (Crispin)	Similar to Golden Delicious, only juicier and coarser
Nicola	Sweet, crisp, juicy
Red Fuji	Sweet, crisp, juicy
Rubinette	Intense honeyed flavor
Salish	Tangy, juicy, very crisp
Topaz	Refreshing, sharp, sweet, mellows with age

**Table 2:** Comparison of survey respondents’ demographics (*N* = 169) with the general British Columbia population.

Item	Category	Survey Respondents (%)	BC Population ^1^ (%)
Gender	Female	59.2	50.4
	Male	29.6	49.6
	Other or unknown	11.2	-
Age (years)	18–25	10.8	9.3
	26–35	22.9	17.4
	36–45	19.3	20.5
	46–55	13.3	20.5
	56–65	16.9	14.7
	66+	16.8	17.6
Ethnicity	Asian, Southeast Asian, and Philippines	15.1	19.0
	European	72.9	63.8
	West Asian and South Asian	6.0	9.1
	Other	6.0	8.1

^1^ Gender, age, and ethnicity statistics for the British Columbia (BC) population were obtained from the Canada 2016 Census data [17]. The age data were approximations, given that the categories used by Statistics Canada (20–24, 25–34, 35–44, 45–54, 55–64, and 65+ years) were slightly different than those used in this study.

**Table 3:** Frequencies of selection of the top four reasons for the purchase of apples by five different classes of customers at the retail and special-event markets.

	Better Eating Quality Seekers	Better Eating Quality Seekers of Familiar or Good-Looking Apples	Taste Lover Buyers	Perfect Product Seekers	Cultivar-Loyal Buyers
**Retail Market**					
Visual appearance	0	0	22.2	100	59.5
Texture	100	72.2	0	100	51.4
Taste/aroma	100	100	100	100	0
Previous experience	0	100	12.1	53.6	32.4
**Special-Event Market**					
Visual appearance	0	100	0	63.6	61.3
Texture	100	88.7	0	85.5	48
Taste/aroma	100	100	100	100	0
Previous experience	0	0	0	100	24

## Supplementary Materials

The following are available online at https://www.mdpi.com/2304-8158/9/7/873/s1, Figure S1: Questionnaire used for evaluation of customers’ behavior, attitudes, and preferences at the UBC Apple Festival.
