# Supplementary figures and images for: Multiple Correspondence and Hierarchical Cluster Analyses for the Profiling of Fresh Apple Customers Using Data from Two Marketplaces
Source: Foods. 2020 Jul 3;9(7):873. doi: 10.3390/foods9070873 (PMC7404775; doi:10.3390/foods9070873)

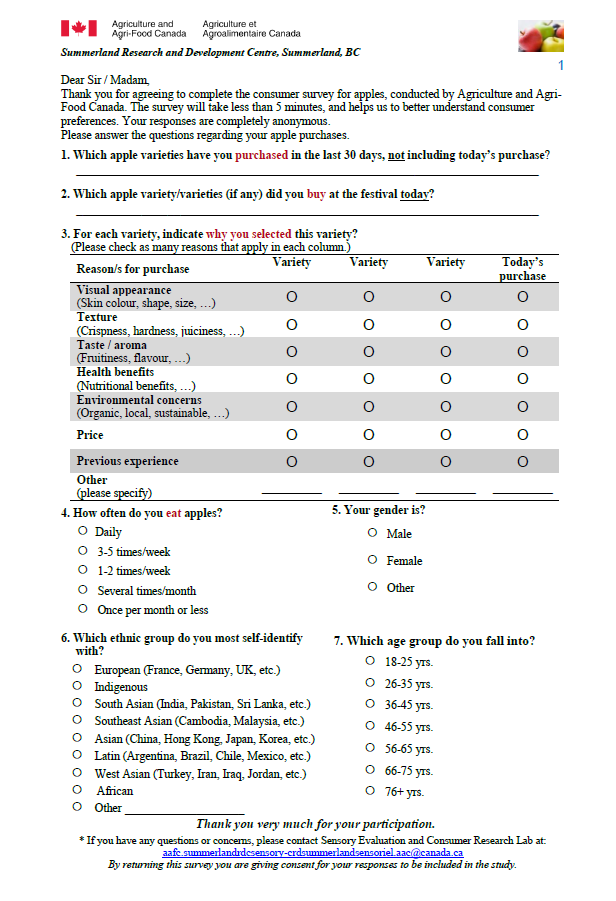

Supplement: Supplementary file 1 [file foods-09-00873-s001.zip › foods-846855-supplementary.tif]
